# Molecular clock in neutral protein evolution

**DOI:** 10.1186/1471-2156-5-25

**Published:** 2004-08-27

**Authors:** Claus O Wilke

**Affiliations:** 1 Keck Graduate Institute of Applied Life Sciences, 535 Watson Drive, Claremont, California 91711, USA; 2 Digital Life Laboratory, California Institute of Technology, Mail Code 136-93, Pasadena, California 91125, USA

## Abstract

**Background:**

A frequent observation in molecular evolution is that amino-acid substitution rates show an index of dispersion (that is, ratio of variance to mean) substantially larger than one. This observation has been termed the overdispersed molecular clock. On the basis of *in silico *protein-evolution experiments, Bastolla and coworkers recently proposed an explanation for this observation: Proteins drift in neutral space, and can temporarily get trapped in regions of substantially reduced neutrality. In these regions, substitution rates are suppressed, which results in an overall substitution process that is not Poissonian. However, the simulation method of Bastolla et al. is representative only for cases in which the product of mutation rate *μ *and population size *N*_e _is small. How the substitution process behaves when *μN*_e _is large is not known.

**Results:**

Here, I study the behavior of the molecular clock in *in silico *protein evolution as a function of mutation rate and population size. I find that the index of dispersion decays with increasing *μN*_e_, and approaches 1 for large *μN*_e _. This observation can be explained with the selective pressure for mutational robustness, which is effective when *μN*_e _is large. This pressure keeps the population out of low-neutrality traps, and thus steadies the ticking of the molecular clock.

**Conclusions:**

The molecular clock in neutral protein evolution can fall into two distinct regimes, a strongly overdispersed one for small *μN*_e_, and a mostly Poissonian one for large *μN*_e_. The former is relevant for the majority of organisms in the plant and animal kingdom, and the latter may be relevant for RNA viruses.

## Background

Kimura has argued that the majority of nucleotide substitutions that accumulate in genes over time are selectively neutral, and go to fixation purely by chance [1]. One major prediction of Kimura's neutral theory is that the substitution process should be a Poisson process, with the mean number of substitutions per unit time equal to the variance. In contrast to this theory, empirical studies often find the variance to be significantly larger than the mean [2-8]. This observation has been termed the "overdispersed molecular clock". (For an excellent review of both empirical evidence and mathematical theories, see Ref. [9].) It is possible to reconcile Kimura's theory with the overdispersed molecular clock via Takahata's fluctuating neutral space model [10-12]. If the neutral mutation rate fluctuates slowly, then the substitution process ceases to be Poissonian, and becomes indeed overdispersed. However, the problem with the fluctuating neutral space model is that it does not offer any argument for why the neutral mutation rate should fluctuate, and thus ultimately fails to explain the observed substitution patterns.

An explanation for fluctuations in neutral mutation rate was recently proposed by Bastolla et al. [13-16]. Different proteins with identical structure naturally vary in their neutrality, that is, in the fraction of single-point mutants that are viable. Therefore, as a gene slowly drifts through sequence space, the neutral mutation rate will fluctuate in correlation to the changing neutrality, and this fluctuation alone could be sufficient to explain the overdispersed molecular clock. Bastolla et al. studied the substitution process in a variety of models of neutral protein evolution *in silico*, and found significant overdispersion in all cases they considered.

However, the simulations that Bastolla et al. carried out were limited to cases in which the product of mutation rate *μ *and population size *N*_e _is small (because Bastolla et al. used only a single sequence as the representative of the whole population, an approach that is justified for *μN*_e _≲ 1). Since population size and mutation rate can be substantial in some species (most notably in RNA viruses), it is justified to ask how general this result is for arbitrary values of *μN*_e_. It is known that large populations and populations evolving under high mutation pressure experience a strong selective pressure to avoid regions of low neutrality, an effect that has been termed "evolution of mutational robustness" [17-20]. In equilibrium, such populations settle in areas of sequence space that have above-average neutrality. As a result, regions of low neutrality are not represented in the population, and the distribution of neutralities in the population is much narrower than the total distribution of neutralities in sequence space. Therefore, we should expect that the neutral mutation rate does not fluctuate strongly under these conditions, and that the molecular clock will not be significantly overdispersed.

For the present paper, I have studied the behavior of the substitution process under neutral protein evolution as a function of mutation rate *μ *and population size *N*_e_. I have found that the accumulation of non-synonymous mutations is substantially overdispersed for small *μN*_e_, in agreement with the results of Bastolla et al., but approaches a Poisson process when *μN*_e _≫ 1. The accumulation of synonymous substitutions is always Poissonian, regardless of the value of *μN*_e_.

## Results

I carried out simulations with DNA sequences of length *L *= 75. I determined the fitness of a DNA sequence by translating it into the corresponding amino-acid sequence, and determining its native fold within the framework of a lattice-protein model. (A sequence would have fitness 1 if it folded into a pre-determined target structure, and fitness 0 otherwise.) I used a simple model of maximally compact proteins on a 5 × 5 lattice. This protein-folding model is much simpler than the ones used by Bastolla et al. [13,14], but has been shown to produce realistic distributions of folding free energies and neutralities [21-23]. The advantage of the simpler model is that entire populations of evolving sequences can be simulated, instead of just individual sequences.

First, I have found that my model produces overdispersion (that is, an index of dispersion *R *substantially above 1) for non-synonymous substitutions, but not for synonymous substitutions. The finding for synonymous mutations is not surprising, because changes in the protein's neutrality do not affect the probability with which a synonymous mutation is neutral (which is always one). Neutral evolution could produce overdispersion in the synonymous substitutions only if the number of synonymous sites in the sequence were undergoing significant fluctuations. While these fluctuations do occur, they are apparently not large enough to affect the index of dispersion.

**Figure 1:**
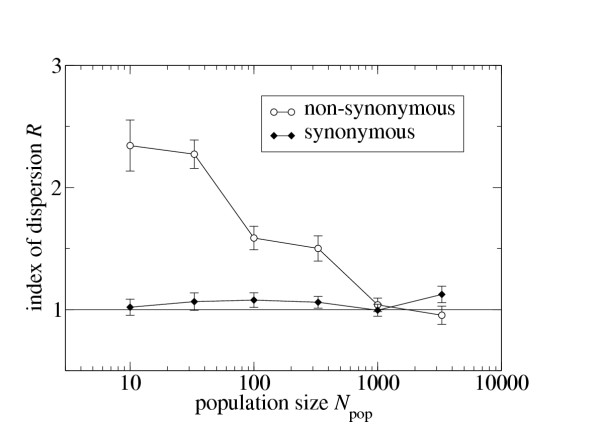
Index of dispersion as a function of population size *N*_e _for synonymous and non-synonymous substitutions (*τ *= 1000, *μ *= 0.075).

**Figure 2:**
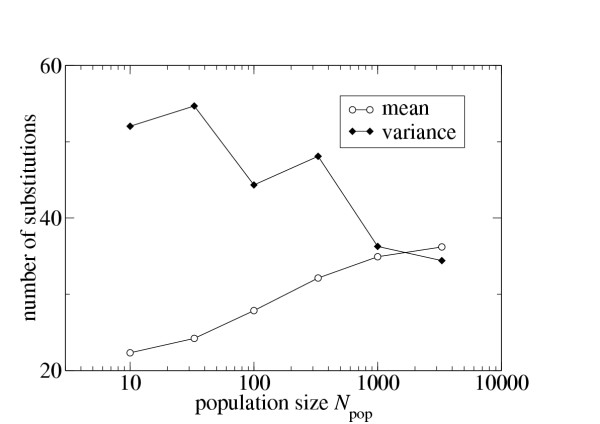
Mean,  and variance,  of lineage-adjusted number of non-synonymous substitutions as a function of population size *N*_e _(*τ *= 1000, *μ *= 0.075). Quantities were calculated from all 500 replicates at each population size.

Second, I have found that for non-synonymous substitutions, *R *decays quickly with increasing population size *N*_e _at fixed *μ *(Fig. 1). Since one reason for a decaying index of dispersion could be a reduced number of accumulated mutations, I have studied how the mean number of accumulated mutations behaves as a function of population size. Instead of staying constant or decreasing, the mean increases with increasing *N*_e_, while the variance decreases (Fig. 2). This result shows that the reduction in *R *is not caused by a mere reduction in the accumulated mutations, and that the substitution process does indeed shift from overdispersed to Poissonian as the population size increases.

**Figure 3:**
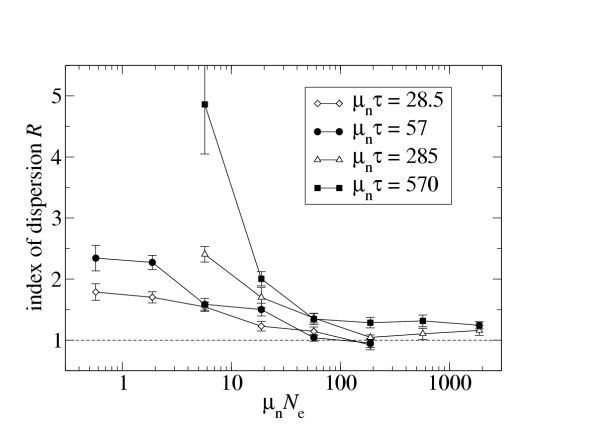
Index of dispersion for non-synonymous mutations as a function of the product of non-synonymous mutation rate *μ*_n _(= 0.76 *μ*) and population size *N*_e_.

For non-synonymous substitutions, *R *decays with *N*_e _because of evolution of mutational robustness. However, mutational robustness is caused by large *μN*_e_, rather than large *N*_e _alone, and the parameter region in which mutational robustness becomes relevant is *μN*_e _≳ 10 [17]. Therefore, it is more instructive to plot *R *as a function of *μN*_e_. The only problem with a naive plot of that sort is that *R *increases as a function of *μτ*, where *τ *is the length of the time window during which mutations accumulate [9]. Thus, in Fig. 3, I show *R *for constant *μτ *as a function of *μN*_e_. Note that in this figure, instead of the sequence-wide mutation rate *μ*, I use the non-synonymous mutation rate *μ*_n _= 0.76 *μ*, which is corrected for the fact that only approximately 76% of mutations hit non-synonymous sites. (76% is the expected fraction of non-synonymous sites in a random DNA sequence.) Figure 3 shows that the transition from an overdispersed to a Poissonian substitution process occurs for *μN*_e _between approximately 10 and 100, in agreement with Ref. [17], and that the transition region seems to be largely independent of the value of *μτ*.

**Figure 4:**
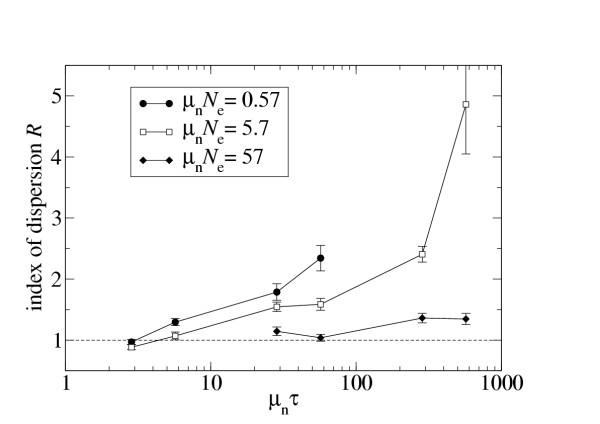
Index of dispersion for non-synonymous mutations as a function of the product of non-synonymous mutation rate *μ*_n _(= 0.76 *μ*) and divergence time *τ*.

Figure 3 also shows that *R *increases with *μτ*. This dependency becomes clearer in Fig. 4, where I display *R *as a function of *μτ *for fixed *μN*_e_. The figure shows substantial increase in *R *with increasing *μτ *for small to moderate *μN*_e_. However, even for *μN*_e _well above 50, there is still a slight increase in *R *with *μτ*. Therefore, my results do not settle the question of whether the substitution process becomes truly Poissonian for sufficiently large *μN*_e_, or whether it just approaches a Poisson process but always remains slightly overdispersed. To settle this question, one would have to carry out simulations with much larger *τ *and *N*_e_. Unfortunately, the protein folding model I use is still too computationally intensive to permit such simulations with current computational resources.

## Discussion

My results show that the size of the product *μN*_e _has a substantial effect on the index of dispersion under neutral evolution. The substitution process is strongly overdispersed for small *μN*e, but approaches a Poisson process as *μN*_e _grows large. Therefore, the next question is which of the two regimes has more biological relevance. As discussed by Cutler [9], the biggest problem in explaining the overdispersed molecular clock is not to come up with mechanisms that produce overdispersion, but to find a general mechanism that does not depend on special conditions or finely-tuned parameters.

To assess the likelihood that fluctuations in neutrality contribute to the overdispersed molecular clock, we have to know the mutation rate and population size for the species of interest. It is notoriously difficult to obtain accurate data for these parameters, and only a few species have been studied in depth. One of the best data sets available is probably the one for *Drosophila*. Keightley and Eyre-Walker estimated the per-nucleotide substitution rate in *Drosophila *to be *u *= 2.2 × 10^-9 ^[24]. If we assume that the average gene in *Drosophila *is 1770 bp long [24], and that 76% of the nucleotides are non-synonymous (this number stems from averaging the number of non-synonymous sites over all codons with equal weight), then the average number of non-synonymous sites per gene is 1345 bp. Thus, the average rate of non-synonymous mutations per gene is *μ*_n _= 3.0 × 10^-6^. With an effective population size of approximately 3 × 10^5 ^[25], we get a product of population size and per-gene-non-synonymous mutation rate of approximately 1. Since selection for mutational robustness starts to take effect when this product is substantially larger than 1, *Drosophila *lies well within the parameter region in which we expect overdispersion to be caused by neutral evolution. For other higher organisms, in particular mammals, which tend to have comparatively small population sizes, we can expect that the product *μ*_n_*N*_e _falls into the same parameter region. On the other hand, for microorganisms, which can have very large population sizes, mutational robustness may play a role in their evolution. In particular, RNA viruses have genomic mutation rates on the order of one [26,27] and their genomes consist typically of only a handful of genes. Because RNA viruses undergo severe bottlenecks on a regular basis, their effective population size *N*_e _is much smaller than the number of virus particles in infected individuals (which can exceed 10^12^), and is more closely related to the number of infected individuals. For HIV-1, *N*_e _has been estimated to be approximately 10^2 ^for subtype A, and 10^5 ^for subtype B [28].

The preceeding paragraph shows that neutral evolution of proteins is probably one source of overdispersed non-synonymous substitutions in *Drosophila *and other organisms. However, overdispersion has been observed in synonymous substitutions as well. For example, Zeng et al. [29] found an index of dispersion *R *significantly above one for synonymous, but not for non-synonymous substitutions in *Drosophila*. For mammals, some studies found *R *significantly above one for both synonymous and non-synonymous substitutions [8], while others found only the non-synonymous substitution process to be overdispersed [30]. Therefore, it is likely that other processes than neutral protein evolution also contribute to overdispersion. Such processes can be selection for optimal codon usage in the case of synonymous mutations, and positive selection on the amino acid level in the case of non-synonymous mutations.

I have demonstrated that large *μN*_e _results in a substitution process with little overdispersion. However, I have not yet given an explanation for how overdispersion is reduced in populations with large *μN*_e_. There are two elements: First, selection for mutational robustness reduces the fraction of sequences with low neutrality, and increases the fraction of sequences with high neutrality, thus making the population more homogeneous and reducing the overall range of neutralities [17-20]. Second, a sequence with low neutrality will experience a real selective disadvantage in comparison to a sequence with high neutrality for large *μN*_e_, and will therefore have a reduced probability to end up on the line of descent. While this selective disadvantage is often small, it can nevertheless determine the evolutionary fate of a sequence in a large population. The larger the population, the more sensitive it becomes to small fitness differences, so that in a very large population a sequence with only a moderate reduction in neutrality will have a small probability to end up on the line of descent. (The fact that the mean substitution rate increases with the population size, as seen in Fig. 2, is also consistent with this reasoning. The larger the population size, the more high-neutrality sequences end up on the line of descent, which is reflected in the increase in the mean substitution rate.)

Throughout this paper, I have considered only neutral or lethal mutations. It is a reasonable question to ask if and how deleterious mutations would change my results. The answer is that they probably have only a minor impact, and the less so the larger *N*_e_, unless they are very slightly deleterious. In order to affect the molecular clock, the deleterious mutations must end up on the line of descent, that is, they must go to fixation. The probability of fixation *p*_fix _of deleterious mutants drops exponentially with the population size, *p*_fix _= [1 - exp(2*s*)]/ [1 - exp(2*sN*_e_)], where *s *is the selective disadvantage of the deleterious mutation [31]. Therefore, for reasonable population sizes, only very slightly deleterious mutations can go to fixation and thus affect the molecular clock. This reasoning is independent of the size of *μN*_e_, as long as *N*_e _is large in comparison to *s*.

## Conclusions

The present study supports the following conclusions:

• Neutral drift of proteins can lead to an overdispersed substitution process for non-synonymous mutations, but not for synonymous mutations.

• The amount of overdispersion in the non-synonymous substitution process depends strongly on the product of mutation rate and population size. As this product increases, the substitution process becomes more and more Poissonian. The transition region starts at *μN*_e _≈ 10, and extends to values well above 100.

• It is not clear whether there are any species that have a sufficiently large population size and mutation rate to prevent overdispersion through neutral drift. In *Drosophila*, the product of mutation rate and population size is close to one, which is well below the parameter region in which the substitution process turns Poissonian.

## Methods

### Lattice protein model

I implemented a version of the 5 × 5 lattice protein model put forward *by *Goldstein and coworkers [21-23,32]. In this model, proteins are sequences of *n *= 25 residues that fold into a maximally compact structure on a two-dimensional grid of 5 × 5 lattice points. There are 1081 distinct possible conformations in this model, and the partition function can be evaluated exactly by summing over the contact energies of all distinct conformations.

The contact energy of a conformation *i *is



where  is the contact energy between amino acids  at location *j *and  at location *k *in the sequence, and  is 1 if the two amino acids are in contact in conformation *k*, and 0 otherwise. The partition function is



where the sum runs over all 1081 conformations. A sequence folds into conformation *f *if the contact energy for that conformation is lower than the contact energies of all other formations, *E*_*f *_<*E*_*i *_for all *i ≠ f*, and if the free energy of folding, which is defined as



is smaller than some cutoff Δ*G*_cut_. Throughout this study, I used *kT *= 0.6 and Δ*G*_cut _= 0. The contact energies  where taken from Table VI in Ref. [33].

### Sequence evolution

I simulated the evolution of populations of DNA sequences in discrete, non-overlapping generations. Population size is denoted by *N*_e_. The fitness of a sequence was 1 if the DNA sequence translated into a peptide sequence that could fold into a chosen target structure, and had a free energy of folding smaller than *G*_cut_. Otherwise, the fitness of the sequence was 0. All sequences had length *L *= 75. In each successive generation, sequences with fitness 1 were randomly chosen to reproduce, until the new generation had *N*_e _members. At reproduction, the sequences were mutated, with an average of *μ *base pair substitutions per sequence. I let each population evolve for several thousand generations, and kept track of the full genealogic information of all sequences in the population. In order to measure the molecular clock of fixed mutations only, I studied the pattern of base substitutions in a window of *τ *generations along the line of descent backwards in time, starting from the most recent common ancestor of the final population.

I varied the parameters *N*_e _(10, 33, 100, 330, 1000, 3300), *μ* (0.0075, 0.075, 0.75), and *τ *(500, 1000). For each set of parameters, I carried out 500 replicates (each with a different, randomly chosen target structure), to obtain a distribution for the number of synonymous and non-synonymous substitutions *S*_d _and *N*_d_. Since there was some variation in the number of synonymous and non-synonymous sites across different target structures (on the order of approximately ± 5% variation from the mean), I then applied a correction factor to *S*_d _and *N*_d _to bring them into comparable units: I calculated the corrected number of synonymous substitutions  as  Here, *S *is the mean number of synonymous sites for the given replicate, and (*S*) is the average of *S *over all 500 replicates. Likewise, I calculated  (Indices of dispersion calculated without this correction factor are slightly larger than the ones reported here, because the variation in *S *and *N *creates additional variance in *S*_d _and *N*_d_). Similar correction factors have been used in sequence analysis [7], and are generally referred to as lineage adjustments. They control for differences among lineages that are primarily related to the expected number of substitutions in a lineage, and thus should not enter the index of dispersion.

To obtain an estimate for mean and standard error of the index of dispersion, I subdivided the 500 results into 10 blocks of 50 each, and calculated mean and variance of the number of substitutions for each block. The ratio of variance to mean for a given set of substitutions (synonymous or non-synonymous) in a block is the index of dispersion for this data set. I then calculated mean and standard error for the index of dispersion from the individual results of the 10 blocks.

The total CPU time needed to carry out all simulations was several months on a small cluster of Pentium II 500 MHz machines.

### Calculation of synonymous and non-synonymous substitutions and sites

I calculated the number of synonymous and non-synonymous sites *S *and *N *and the number of synonymous and non-synonymous substitutions *S*_d _and *N*_d _according to the method proposed by Nei and Gojobori [34]. In short, under this method the number of synonymous sites *s*_*i *_of a codon *i *is the fraction of possible substitutions to that codon that leave the residue unchanged. The number of non-synonymous sites *n*_*i *_for the same codon is *n*_*i *_= 3 - *s*_*i*_. For the complete sequence, *S *and *N *are calculated as  and  where *i *runs over all codons in the sequence. The number of synonymous or non-synonymous substitutions *s*_d,*i *_or *n*_d,*i *_between two codons is the average number of such substitutions, where the average is taken over all paths that lead from one codon to the other. The total number of synonymous or non-synonymous substitutions between two sequences is the sum over all individual constributions,  and  (again, *i *runs over all codons in the sequence).

To calculate the number of synonymous or non-synonymous substitutions along the line of descent, I simply summed up all synonymous or non-synonymous substitutions that occurred from generation to generation. Because the full evolutionary history was known, a correction for multiple mutations such as the Jukes-Cantor correction [35] was not necessary. I also averaged the number of synonymous and non-synonymous sites over all sequences along the line of descent, to get the mean number of synonymous and non-synonymous sites for the given evolutionary trajectory.

## Authors' contributions

COW carried out all aspects of this study.
